# WHAT CONSUMERS SAY ABOUT HOSPICES IN ONLINE REVIEWS

**DOI:** 10.1093/geroni/igz038.3287

**Published:** 2019-11-08

**Authors:** Valeria Cardenas, Anna N Rahman, Mekiayla Singleton, YuJun Zhu, Susan Enguidanos

**Affiliations:** 1 University of Southern California Leonard Davis School of Gerontology, Los Angeles, California, United States; 2 University of Southern California, Los Angeles, California, United States; 3 Leonard Davis School of Gerontology, University of Southern California, Los Angeles, California, United States

## Abstract

Until recently, consumers have had limited resources to assess quality of hospices agencies, contributing to growing numbers of consumers turning to online review sites, such as Yelp. Yet little is known about the content of hospice Yelp reviews and how these relate to recently available Center for Medicare and Medicaid Services’ Hospice Compare site data. No study has examined Yelp hospice reviews and compared the themes identified in Yelp reviews to the topics addressed by CMS’s HC measures. To better understand how consumers perceive hospice care, we drew a purposeful sample of 67 hospices in California. Researchers used grounded theory to identify themes and categories within the hospice reviews. Each of two teams of two researchers independently coded the reviews and then met to compare coding and reconcile discrepancies until 100% consensus was reached. We coded a total of 692 consumer Yelp reviews, identifying 15 themes and grouping them under five overarching thematic categories: patient/caregiver-provider relationship; clinical care; agency competency; staff professionalism; and medical equipment and supplies. We found that overall Yelp comments were positive. The most frequently mentioned Yelp themes in hospice reviews were compassionate, caring staff; patient/family gratitude; and staff responsiveness. There was considerable overlap between the themes captured in HC caregivers survey items and Yelp. However, Yelp reviews cover a greater number and more diverse themes than those measures reported on the CMS Hospice Compare site. We recommend that consumers consider both HC and online review sites such as Yelp when evaluating a hospice.

